# Lag screws for reduction of bilateral lateral mass fractures due to spinal trauma

**DOI:** 10.1016/j.bas.2022.100877

**Published:** 2022-03-12

**Authors:** Massimimiliano Minardi, Alessandro Narducci, Giovanni Giulio Vercelli, Christian Francesco Carlino, Federico Griva, Pier Federico Pretti

**Affiliations:** a Department of Neurosurgery San Giovanni Bosco Hospital, Piazza del Donatore di Sangue 3, Turin, Italy; b Neurosurgery Unit, Department of Neuroscience, Città della salute e della Scienza Torino, Via Cherasco 15, Turin, Italy

## Abstract

**Introduction:**

Bilateral fracture of the C1 lateral mass is a relatively uncommon type of traumatic lesion. Treatment of this kind of fractures is usually conservative, with either external immobilization or traction.

**Research question:**

Whether surgical management, with placement of lag screws in lateral mass of C1, could represent a first-line treatment.

**Material and methods:**

We describe a case of 67-years old man with bilateral fractures of lateral mass of Atlas due to road accident trauma without ligament lesion but severe gap between bone edges. We performed Computed Tomography and Magnetic Resonance scans for pre-operative imaging, X-Ray and CT scan for follow-up. Medtronic navigation system was used as intraoperative guidance for screw placement.

**Results:**

Radiological and clinical results were good, with optimal bone reduction and patient's early return to daily activities.

**Discussion and conclusion:**

Surgical management remains debateable for isolated C1 lateral mass fractures. Different surgical approaches have been described for atlas fractures, such as transoral anterior C1-ring plate osteosynthesis, posterior osteosynthesis with a lateral mass screw rod, and posterior C1 to C2 fusion and C0 to C2 fusion. Minimally invasive operative treatment with lag screw and reduction of fracture's edges without occiput-C1 or C1-C2 stabilization could be the optimal treatment with good result and decreasing rate of pseudoarthrosis, allowing to avoid Halo-vest discomfort and complications.

## Highlights

• Bilateral fracture of the C1 lateral mass is relatively uncommon type of traumatic lesion.
• When gap among fracture's edges is severe there is high rate of not fusion or pseudoarthrosis.
• Surgical reduction with bilateral lag screw, preserving C1-C2 motion and good results at follow-up imaging.

## 1 Introduction

Atlas fractures (AF) represent the second most common injury of upper cervical spine (2–13%) (Kandziora et al., 2017). In most cases, treatment is conservative with collar or external fixation because in the great majority of fractures are stable. Surgical management is mandatory when fractures are unstable due to disruption or incompetency of Transversal Ligament of the Atlas (TLA) (Koller et al., 2010), that represents the most important determinant of C1-C2 stability during cervical motion.

C1 lateral mass fractures (unilateral or bilateral) often are stable injury and, in most cases, treatment is conservative. Notwithstanding, when gap between fracture's edges is severe, with subsequent high risk of fusion failure or pseudoarthrosis, in case of TLA disruption, and when patient does not accept the discomfort of Halo-vest, surgical treatment represents a reasonable option. We describe a case of a successful surgical treatment through bilateral C1 lag screw placement of a patient with bilateral lateral mass fracture, underlying advantages of surgery in this particular and rare type of fractures.

## 2 Case presentation

**Fig. 1:**
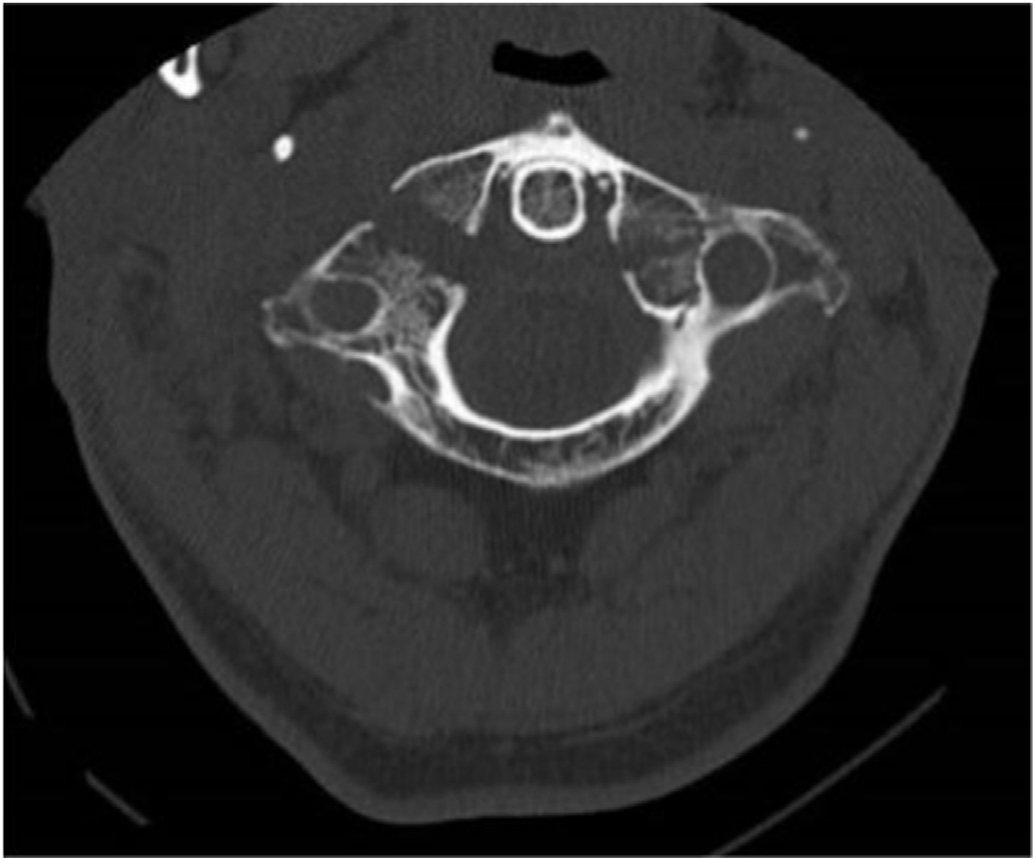
CT scan pre op: demonstrate severe bilateral fractures' edge gap.

**Fig. 2:**
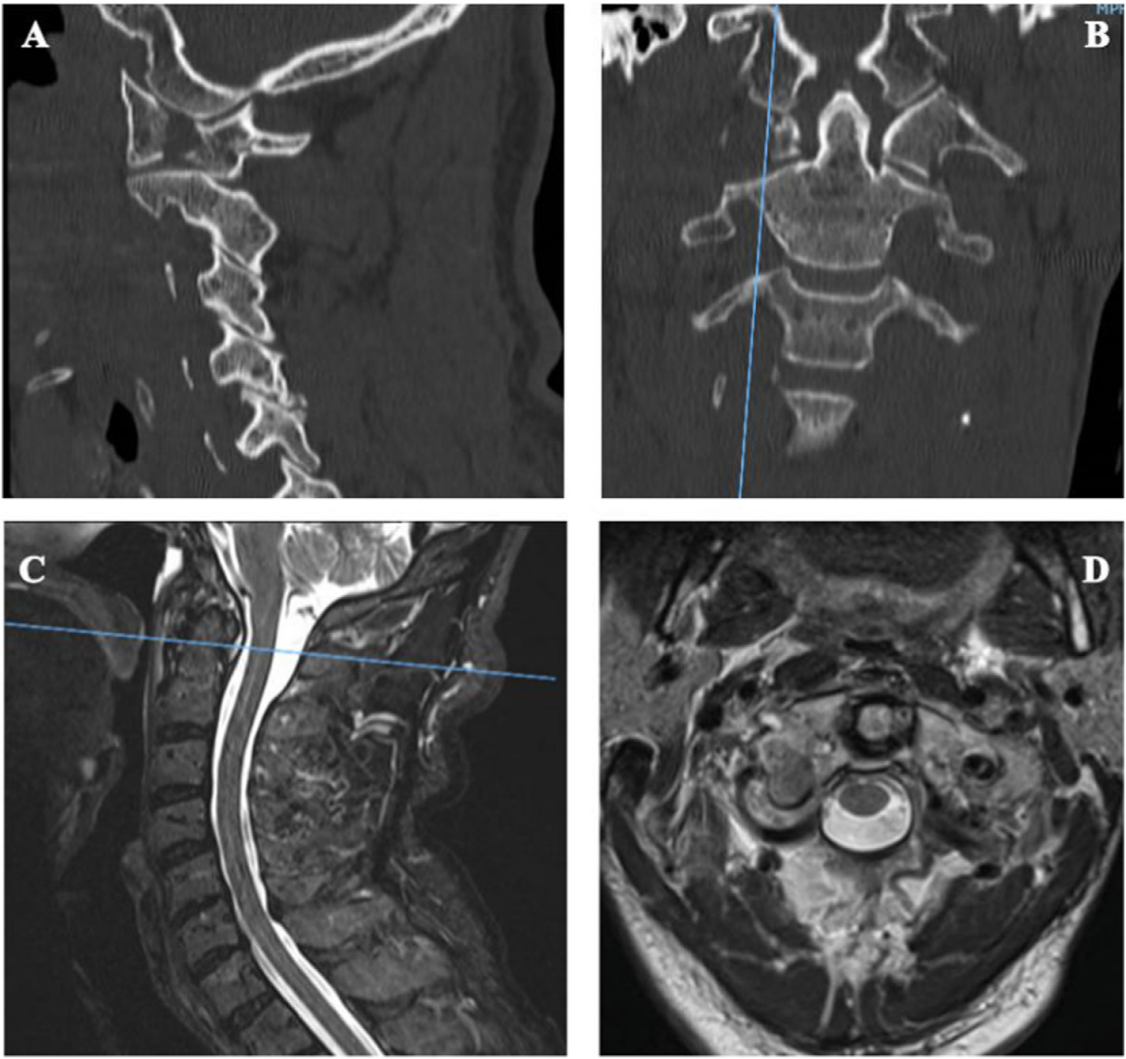
(A–B) CT scan pre-op sagittal and coronal view respectively; (C–D) Cervical-MR: demonstrate no ligament damage.

A 67-year-old man presented to our Emergency Department following a road accident. He was neurologically intact, complaining only upper cervical pain mostly on motion, despite he did not have a clear limitation of cervical range of movement. Urgent cervical CT-scan detected bilateral lateral mass fractures of C1 (type IV according to Gehweiler classification), more severe at right side with gap between the fracture's edges of approximately 8-mm (Fig. 1, Fig. 2) whereas on the left side the space was around 3.5 mm.

Also Magnetic Resonance Scan showed intact cranio-cervical ligaments (Fig. 2). No signs of myelopathy were detected. The patient refused conservative treatment with Halo-vest, so that we decided to perform a direct surgical reduction of the fracture due to the high risk of pseudoarthrosis with hard collar alone.,

## 3 Surgical treatment and follow-up

**Fig. 3:**
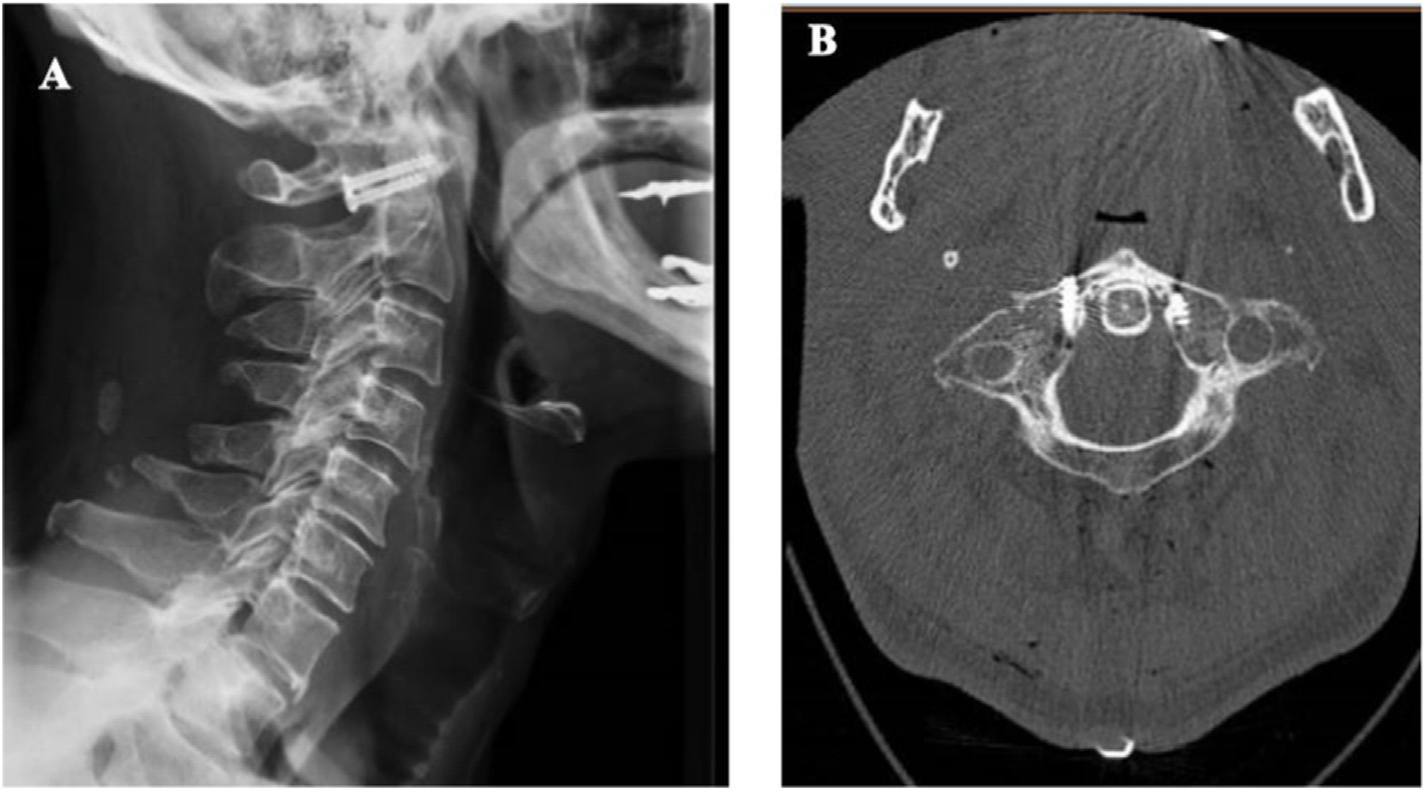
(A–B) RX and CT one day post-op respectively.

**Fig. 4:**
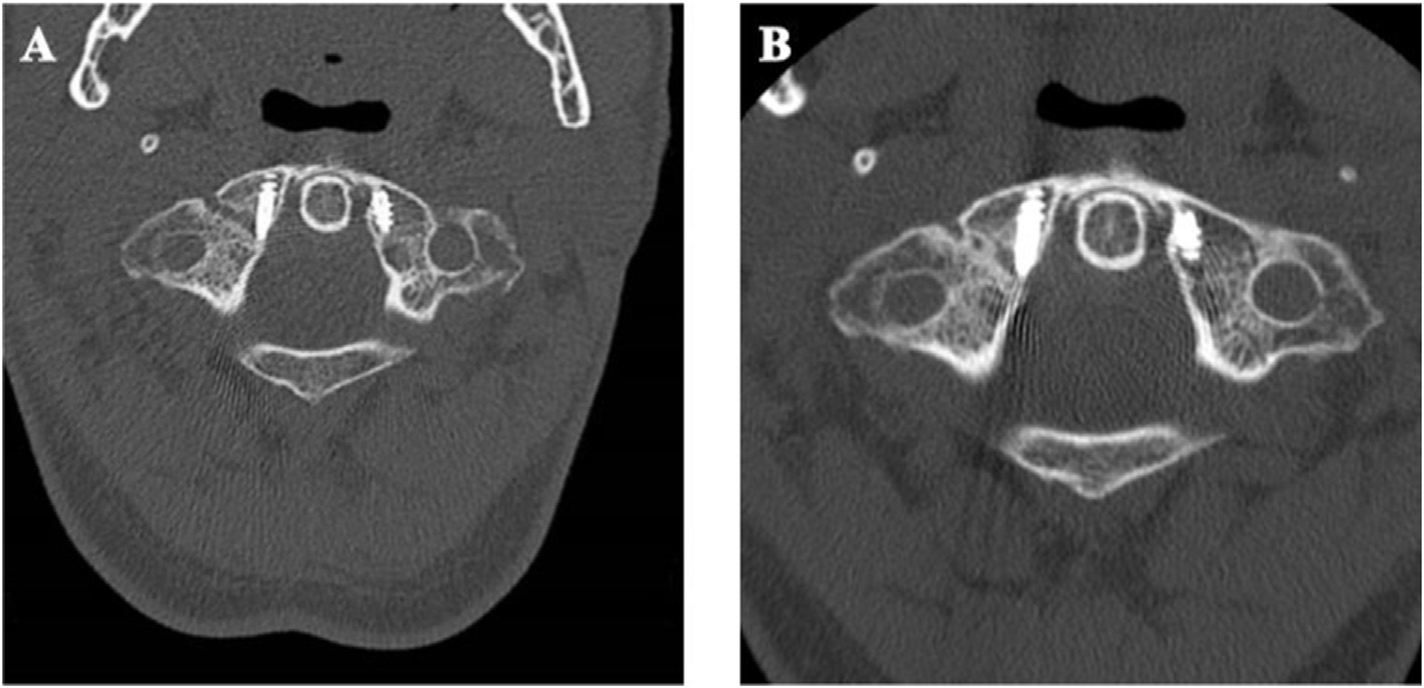
(A–B) CT scan follow up at 3 and 9 months respectively with good reduction and bone edge fusion.

Surgical planning and study of screw trajectories was performed through a Neuronavigation system (Medtronic S8). Patient was in prone position with the head onslight flexion, fixed in radiolucent Mayfield three-pin clamp. Standard midline approach was performed from occiput to C1-C2 joint, without exposure of the posterior arch of C2, in order to reduce cervical muscle dissection. Dissection was continued until visualization of venous perivascular plexus of vertebral artery, identifying C1 lateral mass. Screw entry point was carefully checked with the aid of Navigation system. A 2mm awl high-speed drill was used to mark the entry point. We then proceeded with the guide wire placement. Partially threaded cannulated screws were used; the length for each side was calculated based on the CT scan measurement, adding the length of the two bone stumps to be synthesized. A 22-mm screw was placed on the right side and a 20-mm screw on the left (Mathys, Switzerland) The trajectory was about 15° medial and 25 rostral from the entry point. Intraoperative lateral X-ray images confirmed immediate good reduction The result was confirmed by post-operative CT scan, which showed a near complete bilateral reduction of the fracture.(Fig. 3). Patient was able to walk without any aid on the first postoperative day with cervico-thoracic brace and he was discharged home after two days without any deficit. CT images at three and nine months follow-up, demonstrated total fusion on the left side, and nearly total on the right side (Fig. 4).

## 4 Discussion

The best treatment for isolated C1 fracture, which represent about 2–13% of upper cervical spine lesions, is not well established by (Kandziora et al., 2017). The most common causes of these fractures are motor vehicle accidents, and the mechanism of injury is axial loading. Often, 40%–44% of atlas fractures have concomitant axis fractures. (Kakarla et al., 2010).

C1 fractures are usually stable and treatment is conservative with cervical collar immobilization. However, if associated with atlantoaxial ligamentous (TLA) lesion they may require surgical stabilization due to the instability of craniovertebral junction. The decision making for treatment is not always straightforward, due to the high variability of fracture pattern. Gehweiler classification provides a useful guidance, describing five type of fractures according to the location of damage. Type I, II, and III are fractures of the anterior arch, posterior arch, and anterior and posterior arch (Jefferson burst fracture) respectively, while type IV and V are fractures of the lateral mass and isolated fractures of the C1 transverse process respectively (Gehweiler et al., 1979).

Lateral mass a fractures (LMAF) can be included in type IV of atlas fracture considering the above mentionated classification (Gehweiler et al., 1979), and often are considered stable injuries (Inaoka et al., 2007). Despite that, literature is controversial regarding the best therapeutic approach. Commonly conservative management with cervical collar represents the first option in stable fracture with minimal displacement. HALO-vest immobilization for almost three months is required in case of significant dislocation of the fractured lateral mass. Surgical osteosynthesis should be considered as second option, in case of failure of reduction and fusion with Halo-vest.

In this type of fracture an important factor to consider is the gap between bone edges. This aspect is crucial, because a significant gap could be associated with high rate of fusion failure and pseudoarthrosis (Kakarla et al., 2010; Levine and Edwards, 1991; Patton and Renshaw, 2006), therefore surgery could actually represent the best option.

Many authors reported cases of surgically treated C1 lateral mass fractures (Table 1) (Farrokhi et al., 2019; Felbaum et al., 2018; Gelinas-Phaneuf et al., 2018; Li et al., 2011; Patton and Renshaw, 2006; Zhang et al., 2018), but surgical management remain discussed and different type of surgery have been described, such as transoral approach anterior C1-ring plate osteosynthesis, posterior osteosynthesis with a lateral mass screw rod, and posterior C1 to C2 fusion and C0 to C2 fusion. Posterior C1 to C2 fusion with transpedicular fixation is the most commonly used method for unstable atlas fracture. This procedure has the advantage of safe fixation and high fusion rates, but there are also some drawbacks such as range of motion limitation and the non-negligible risk of vertebral artery injury.

**Table 1:** Review’s Table: illustrate the pregress articles’ of minimal invasive treatment of C1 fractures.

Author's Study and years	Type of study	N. of patient	Type of fracture	Ligament lesion	Symptoms	Management	Follow-up
M.F. Farrokhi et al., 2018	Case report	1	Unilateral	no	No	Minerva orthosis 2 mo then lag screw	18 month good result
D.R. Felbaum et al., 2017	Case series	2	Unilateral	no	Neck-pain	-Halo-vest 2mo then lag screw	6–8month good result
						-Bracing 6mo then lateral mass screw	
R. Bransford et al., 2011	Case series	3	Unilateral	no	No neurological deficit but correlate to other spine injuries and politrauma	Open reduction and internal fixation	-2patients mean follow-up 14,6 mo
						with 2 unilateral transversely oriented lateral mass screws	-1 patient death at 2mo
Tabbosha et al., 2013	Case report	1	Unilateral extended to inferior and superior facet join	no	Neurologically intact. Head and neck pain. Posterior cervical spine tenderness	7 weeks of rigid collar.	10 month: Improvement of neck pain, muscle
						After open reduction and internal fixation	spasms, and cervical range of
						with placement of a unilateral lag screw	motion postoperatively
Li et al., 2011	Case series	2	Unstable Jafferson Fractures	yes	-neck pain, stiffness, and decreased range of motion without neurologic deficit	Direct posteior C1 lateral mass screws compression reduction and osteosynthesis	12 mo good result
Our Study 2021	Case report	1	Bilateral	no	Neck pain high gap of fracture's edge	Primary Lateral mass lag screws bilaterally	

In the case we reported, which is a type IV of Gehweiler, we preferred to perform surgery as first option in order to offer to the patient the best chances of bone reduction, avoiding the important discomfort and complications associated to immobilization with HALO-vest.

We can state that the gap among fracture's edges has represented the most important factor we considered in the decision making. In fact, literature is clear about the risk of fusion failure, pseudoarthrosis, with subsequent deformity in case of severe fracture gap. This is caused by the gap itself, a well-known and intuitive principle, but also by the excessive friction determined by the occipital condile on the lateral C1 mass with bone spaced apart. This can determine a clinical severe course with chronic pain, impairment of cervical motion, and important limitation in daily activities and work (Butler et al., 2010; Meeson et al., 2019; Müller et al., 2003).

Based on the assumptions arising from the case we reported, we could state that Gehweiler type IV fractures are not a unique entity. Fracture orientation and the entity of the gap between the edges have utmost importance in clinical practice; so that we think that they should be sub-classified according with these parameters.

## 5 Conclusion

The best treatment for C1 lateral mass fractures is not well established. In our experience, fracture direction and amount of bone displacement are the main determinants in decision-making. Fracture reduction through lag screws can be considered an effective and safe treatment, since it allows to avoid the discomfort and risks of Halo-vest immobilization, providing in the meantime a high chance of fracture healing.

## Funding

This research did not receive any specific grant from funding agencies in the public, commercial, or not-for-profit sectors.
